# Reconstruction of the Chemical Gas Concentration Distribution Using Partial Convolution-Based Image Inpainting

**DOI:** 10.3390/s24144470

**Published:** 2024-07-10

**Authors:** Minjae Kang, Jungjae Son, Byungheon Lee, Hyunwoo Nam

**Affiliations:** Chem-Bio Technology Center, Agency for Defense Development, Daejeon 34186, Republic of Korea; kmjs0328@add.re.kr (M.K.); jjson0509@gmail.com (J.S.); bhl1126@add.re.kr (B.L.)

**Keywords:** data reconstruction, gas dispersion, image inpainting, spatial interpolation, partial convolution, machine learning, chemical sensing

## Abstract

An interpolation method, which estimates unknown values with constrained information, is based on mathematical calculations. In this study, we addressed interpolation from an image-based perspective and expanded the use of image inpainting to estimate values at unknown points. When chemical gas is dispersed through a chemical attack or terrorism, it is possible to determine the concentration of the gas at each location by utilizing the deployed sensors. By interpolating the concentrations, we can obtain the contours of gas concentration. Accurately distinguishing the contours of a contaminated region from a map enables the optimal response to minimize damage. However, areas with an insufficient number of sensors have less accurate contours than other areas. In order to achieve more accurate contour data, an image inpainting-based method is proposed to enhance reliability by erasing and reconstructing low-accuracy areas in the contour. Partial convolution is used as the machine learning approach for image-inpainting, with the modified loss function for optimization. In order to train the model, we developed a gas diffusion simulation model and generated a gas concentration contour dataset comprising 100,000 contour images. The results of the model were compared to those of Kriging interpolation, one of the conventional spatial interpolation methods, finally demonstrating 13.21% higher accuracy. This suggests that interpolation from an image-based perspective can achieve higher accuracy than numerical interpolation on well-trained data. The proposed method was validated using gas concentration contour data from the verified gas dispersion modeling software Nuclear Biological Chemical Reporting And Modeling System (NBC_RAMS), which was developed by the Agency for Defense Development, South Korea.

## 1. Introduction

**Figure 1:**
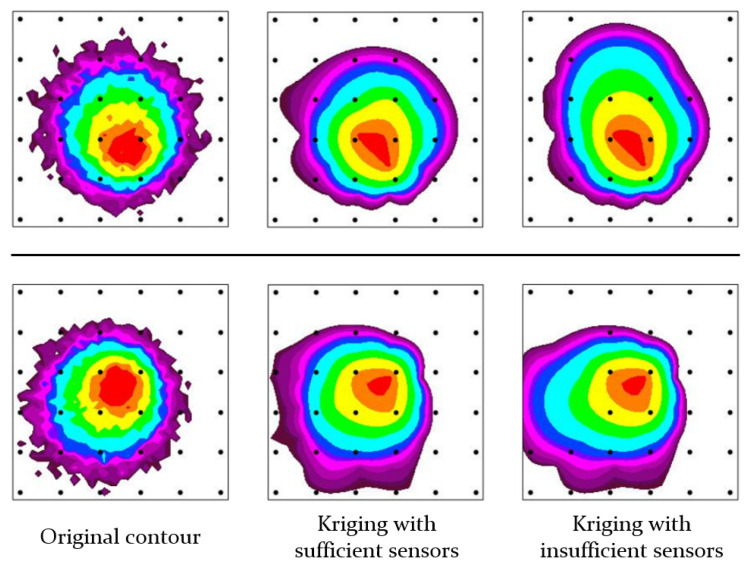
Difference in the reliability of contours according to the number of sensors. The pictures on the left are the original contours obtained using the gas diffusion simulator we developed. The middles are the contours created using Kriging interpolation based on a sufficient number of values sampled from the original contours. The pictures on the right are the contours generated based on an insufficient number of values. The black dots represent the positions of chemical sensors. The concentration data on those points are used for Kriging interpolation.

When chemical gas is dispersed in chemical attacks, terrorism, or safety accidents, the gas forms a plume and diffuses in various weather conditions. Since the diffused gas is colorless and odorless, it can cause severe casualties. It is important to determine the extent and concentration of gases to minimize casualties. Chemical sensors can also be used to assess the spread of gas. It is possible to generate a gas concentration contour using the data measured by the sensors. For a rapid response against gas dispersion, it is advantageous to install a large number of detection sensors. However, resources are limited, and the reliability of contours where sensors are relatively scarce is low. Figure 1 shows the difference in the reliability of contours according to the number of sensors. As shown in the figure, the contours generated by an insufficient number of sensors do not accurately reflect the actual environment. Therefore, we proposed a method for enhancing the reliability of these areas by reconstructing inaccurate regions using image inpainting.

Mathematical methods have commonly been used to estimate data for unknown areas using limited information. Spatial interpolation is one of the mathematical estimation methods. The input contour image for our method was also generated using spatial interpolation, and unreliable regions within the image were reconstructed using image inpainting. Unlike other studies aimed at improving the accuracy of interpolation from a mathematical perspective, we addressed the same problem from an image-based perspective.

Recent approaches [1,2,3] to image inpainting are based on generative models such as generative adversarial network (GAN) [4], transformer [5,6,7], and denoising diffusion probabilistic model (DDPM) [8]. These methods demonstrate effective performance in plausible filling by utilizing the strengths of the image processing of each model. However, generative model-based approaches generate only plausible and diverse images of missing regions. Filling the missing region plausibly is not meaningful in this task because the purpose of the reconstruction of concentration contours is to improve accuracy. In this study, a partial convolution [9]—based model was proposed among the various models of image inpainting. Since the gas concentration is continuous and mathematically highly correlated with nearby values, it would be more proper to fill it using nearby values rather than simply filling it plausibly. In contrast to generative models, partial convolution fills in missing regions using the convolution calculation of nearby valid regions. Additionally, image inpainting using partial convolution is robust, even with irregular missing data, allowing for more flexible and rational contour reconstruction. By adding a new loss term and tuning the coefficients of the loss function in [9], the model demonstrated better performance in gas concentration contour reconstruction.

In order to train the model, a contour dataset representing the concentration of a dispersed gas is required. Obtaining a sufficient amount of actual data is restricted by time costs and economic constraints. Therefore, a gas diffusion simulation model was developed to construct the concentration contour dataset. This dataset consisted of contour images, and the simulator was based on a two-dimensional Gaussian distribution. This diffusion simulation model also considered wind effects and particle viscosity to generate realistic image data. After training, the utility of the model was validated using the Nuclear Biological Chemical Reporting And Modeling System (NBC_RAMS) developed by the Agency for Defense Development (ADD), South Korea. NBC_RAMS is capable of utilizing validated mathematical models to simulate the behavior of gas particles accurately. The validation was conducted by comparing the results of the software with those of the reconstructed contours using the proposed model. Consequently, we demonstrated that image inpainting can improve the accuracy of the spatial interpolation of gas concentration distribution.

The main contributions of this paper can be summarized as follows:

- The proposed model, optimized for gas concentration reconstruction, demonstrates an accuracy that is 13.21% higher than that of conventional spatial interpolation methods. This improvement in accuracy has been validated using reliable software, NBC_RAMS;
- The enhanced accuracy in identifying gas diffusion allows for a rapid response in scenarios involving the leakage of hazardous gases, thereby enabling prompt action in emergencies to prevent significant casualties;
- The proposed model improves economic efficiency. In real-world scenarios where the deployment of numerous sensors is impractical, it enables effective situation assessment using a limited number of sensors. Additionally, the model can be applied to various fields involving spatially distributed data.

## 2. Related Works

### 2.1. Image Inpainting

Image inpainting is a technique used to plausibly fill an erased space in an image; it is often used to restore the corresponding position after removing specific objects. In its early days, nonlearning methods were proposed and were mainly categorized into diffusion- and patch-based methods. Diffusion methods propagate the information of the valid image regions surrounding the erased area to fill them in. Bertalmio et al. [10] and Ballester et al. [11] introduced the early diffusion method using anisotropy. Chan et al. [12] later modified the total variation denoising model for the inpainting of nontextual images. Other methods introduced by Demanet et al. [13] and Levin et al. [14] use image statistics for diffusion-based image inpainting. While diffusion methods excel at filling small or narrow areas, they struggle with large areas. Patch-based methods operate under the assumption of redundancy and coherence in natural images, where nearby pixels tend to share similar characteristics. Many methods have been proposed to search for patches to fill the erased space. Examples include using structure-based priority for patch searches [15], using Markov random fields [16], and applying nonlocal texture matching and nonlinear filtering [17]. However, these methods also have limitations in effectively filling larger areas.

As deep learning has shown significant potential in image processing, many methods using deep learning techniques have emerged in the field of image inpainting. Specifically, convolutional neural networks (CNNs) and generative models have been widely used for image inpainting tasks. Pathak et al. [18] applied a CNN-based autoencoder architecture to learn the mapping between an input image with missing regions and a complete image. The partial convolution approach [9] fills in the image using a convolution operation only on the valid pixels around the missing region. Yu et al. [19] divided the inpainting process into two steps and utilized gated convolution. Various methods for generating images corresponding to erased regions have been developed using GANs, transformers, and DDPMs. Notably, approaches such as those using adversarial and contextual losses with a conditional GAN [20], as well as methods using CNNs and GANs to predict edge guidance in erased areas for inpainting [21], have been proposed. The method proposed by Wan et al. [3] uses a transformer and CNN to decouple image inpainting into an image structure recovery step (for the transformer) and a resolution upsampling step (for the CNN). Lugmayr et al. utilized DDPM to complete an image using sampled noise, which is a process applicable regardless of the mask shape [2].

### 2.2. Spatial Interpolation

Spatial interpolation techniques are used to estimate values at unmeasured locations based on known data. Representative methods such as inverse distance weighted (IDW), Kriging, and splines have been extensively compared in many studies.

IDW interpolates by forming a linear combination based on data from unknown regions. Compared to other methods, it has the advantage of being simple and less computational but has the disadvantage of being sensitive to outliers. The Kriging method, similar to IDW, estimates data at an unmeasured location but incorporates a variogram, which measures the spatial correlation between two points and contributes significantly to the Kriging method’s high accuracy. While it offers high accuracy, the Kriging method demands significant computational resources. The spline method performs interpolation using a mathematical function and estimates by fitting the known data into a spline function. It has the advantage of being flexible for complex patterns; however, it has low reliability towards the edge of the estimation region.

Although the suitability of the method differs depending on the specific geographic area and the aspect of data existence, many studies have reported that the accuracy of the Kriging method is high in many fields. Bekele et al. [22] compared the performances of the Kriging method and IDW and demonstrated that the Kriging method generally has high performance. Laslett et al. [23] similarly compared the Kriging and spline methods and demonstrated that Kriging has better accuracy.

## 3. Materials and Methods

### 3.1. Dataset

**Figure 2:**
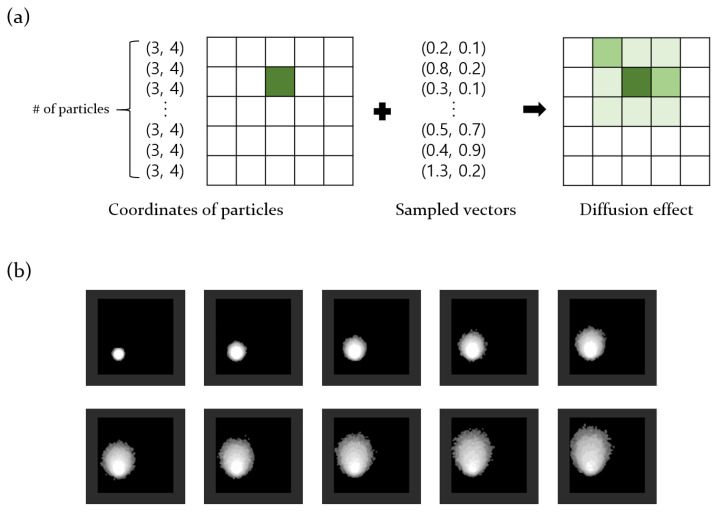
(**a**) Gas diffusion process in the developed simulator. Before diffusion, all gas particles are located at specific co-ordinates. However, as sampled vectors are added, the particles move, resulting in diffusion, as shown in the grid map on the right. (**b**) Result of the diffusion process.

The accuracy of the model in real-world situations depends on how closely the dataset reflects actual environmental conditions. Generating a dataset that mimics real-world situations requires access to sensor-measured concentration values. However, gathering extensive data by dispersing gases in an actual environment is both economically and time-intensive. In order to overcome this challenge, we developed a simulation model that emulates gas diffusion. In this simulation model, gas diffusion follows a two-dimensional Gaussian distribution. Figure 2 illustrates the diffusion process.

**Figure 3:**
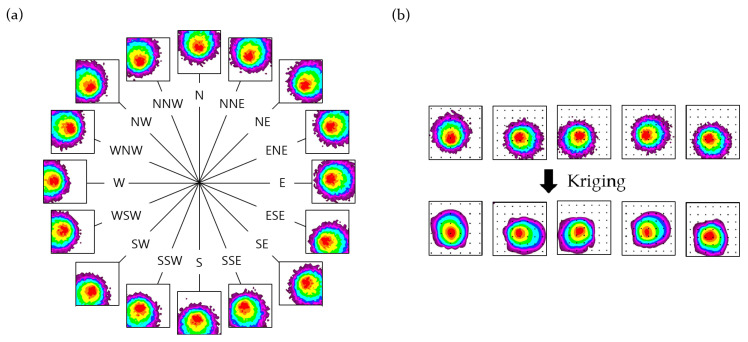
(**a**) The results of the gas diffusion simulation model. The shape of the gas diffusion contour varies depending on the wind blowing in 16 directions. (**b**) The portion of the dataset created from the simulation results. The images below were created using the Kriging method, using 49 concentration points indicated by the black dots in the simulation results above.

Gas diffusion in the simulation model occurred on a two-dimensional grid map, and each gas particle stored the two-dimensional co-ordinate vector of the current position. Once the origin grid of gas diffusion was set, all particle vectors were set in that grid co-ordinate. Subsequently, an equal number of two-dimensional random vectors were sampled from a Gaussian distribution. By adding these sampled vectors to the co-ordinate vectors of the gas particles, each particle moved on the grid, facilitating diffusion. The gas diffusion simulation model also considers the effects of wind. The wind effects were categorized into 16 directions, with corresponding vectors for each direction added to all particles at regular intervals. In reality, wind affects gas particles differently based on concentration. High-concentration areas are less affected by wind compared to low-concentration areas. In order to reflect this viscosity of particles, we multiplied a value from 0.5 to 1 for each wind vector according to the concentration of each grid before adding it. Consequently, a small-magnitude wind vector was added to the co-ordinate vector of the gas particles located in areas with high concentrations. Figure 3a shows the results of the gas diffusion simulation model based on 16-directional winds.

**Figure 4:**
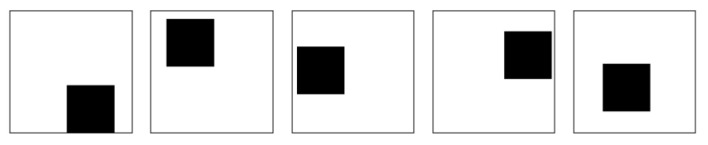
The portion of the masks for training. Black rectangles are used to mask the different regions of the training data, with their positions varying randomly for each mask. This process removes random areas of the training data, enabling the model to learn from a diverse set of scenarios.

Each image within the dataset was 256 × 256 pixels and was created using the Kriging method, utilizing 49 concentration points from the results of the gas diffusion simulation model. Figure 3b shows a segment of the gas concentration contour dataset created from the simulation results. We also created 100 masks for training. Each mask image has a randomly masked region with a size of 100 × 100 pixels, which is about 15% of the input image. The mask was filled with zeros in the missing regions and ones in the valid regions. Figure 4 shows samples of the mask images.

### 3.2. Partial Convolution

Partial convolution was adopted to reconstruct the low-reliability areas of the contour. Partial convolution, proposed by Liu et al. in 2018 [9], is an image-inpainting method that utilizes a CNN. In this approach, unlike other convolution-based methods, the convolution filter slides across the image and performs convolution operations solely on the valid pixels not covered by a mask. Consequently, the missing regions of the images are filled in as a result of the operation. In addition, the masks are updated gradually after each partial convolution operation. These differences enable partial convolution-based inpainting to effectively handle irregularly sized masked regions. The update process for the images and masks can be expressed as

(1) $$x^{\prime }=\left\{\begin{array}{cc} W^{T}\left(X⊚M\right)\frac{sum(1)}{sum(M)}+b & \mathrm{if}\;sum(M)>0\\ 0 & \mathrm{otherwise}. \end{array}\right.$$

(2) $$m^{\prime }=\left\{\begin{array}{cc} 1 & \mathrm{if}\;sum(M)>0\\ 0 & \mathrm{otherwise}. \end{array}\right.$$

where **W** is the convolutional filter, **X** is the image feature corresponding to the filter’s location, and **M** is the corresponding feature of the binary mask. **1** has the same shape as the filter, with all the elements being 1, and it is used for calculating the scaling factor. When the filter slides, if there are nonzero pixels in the image, the corresponding regions of the image and mask are updated. Nonzero pixels represent valid regions that were not erased by the mask. As the image passes through the partial convolution layers, all the missing regions in the image and mask are gradually filled progressively from the valid regions.

#### 3.2.1. Network

The proposed model has a UNet-like architecture network [24] composed of partial convolution layers. The encoding layers use convolution operations and activation functions that reduce the dimensions of the image, and the decoding layers use nearest neighbor upsampling to expand the image dimensions. The features extracted from the image during the encoding stage were passed to the decoding layers through the skip links of the UNet architecture. These features were concatenated with the results of the previous decoding block and then forwarded to the next layer. Equations (1) and (2) were applied to the convolution operations in the encoding stage to update the input for the next layer. Consequently, the missing regions in the image are filled, and the mask is updated during encoding. By using the features obtained through the skip links from the encoding stage, the decoding layers can obtain information regarding the missing regions of the image. The U-Net architecture’s ability to effectively extract gas diffusion patterns in the encoding layers and fully utilize the input contour information in the final layer is advantageous for gas contour reconstruction.

#### 3.2.2. Loss Function

The loss function of partial convolution [9] was adjusted for the proposed model’s performance. The coefficients of each loss term were modified, and a new term was added to better fit the model to the task of contour reconstruction. The loss function includes newly defined perceptual loss, two style losses [9], and the total variation loss proposed by Johnson et al. [25]. Additionally, the loss function also includes two $L_{1}$ loss terms that compare the masked and unmasked regions with the ground truth. ImageNet-pretrained VGG-16 [26] was used to calculate the perceptual loss and style losses. In order to improve the model’s performance, an SSIM (structural similarity index) [27] loss term was added. SSIM is frequently utilized in learning methods that aim to make images similar since it represents structural similarity at the pixel level between two images. The total loss function consisting of seven terms can be represented as Equation (3), and the results were compared according to various tuning conditions.

(3) $$\begin{array}{c} Loss=L_{vaild}+21L_{hole}+0.05L_{perceptual}+30\left(L_{style_{out}}+L_{style_{comp}}\right)+0.1L_{tv}+L_{ssim}\;. \end{array}$$

### 3.3. Loss Tuning

The contour images reconstructed using this model are represented by discrete colors, where each color represents the gas concentration. Since pixel smoothing is not crucial, the coefficients of the two $L_{style}$ were reduced to allow the loss to converge better. In addition, due to the critical importance of accuracy in the filled regions for this task, the coefficient of $L_{hole}$, which represents the loss of the filled areas, was increased. As the model is trained to minimize the loss, modifying these coefficients and adding the SSIM loss term helps the model focus more on accurately filling the interior regions to match the training data. We performed a hyperparameter search to determine the coefficients of the loss function, and the tuned model demonstrated a higher mIoU score of 3.24%. The score was calculated only for the regions filled in by the model. The mIoU score can be calculated using Equation (4). OverlapN indicates the number of pixels overlapping with the ground truth for each class, where each class represents 13 different colors for contour representation. The increase in mIoU score through loss tuning demonstrates that the model has become more suitable for gas contour reconstruction.

(4) $$mIoU=\frac{Overlap1+Overlap2+\cdots +Overlap13}{number\;of\;pixels\times number\;of\;classes}\;.$$

## 4. Results

### 4.1. Training Process

**Figure 5:**
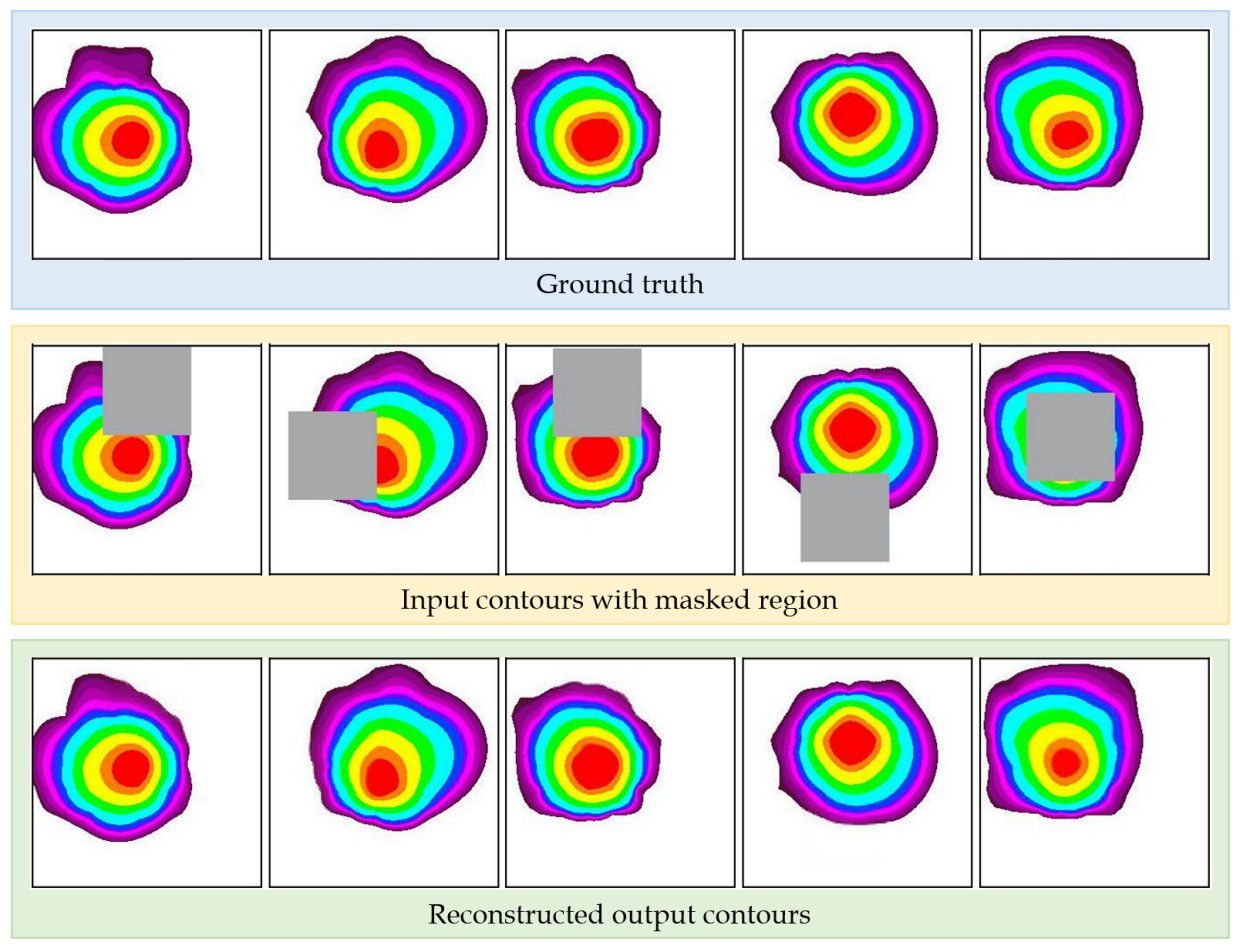
Training results. The pictures in the blue box are part of the test dataset. The reconstructed results of the model for randomly masked input images are presented in the green box.

In order to train the contour reconstruction model, we utilized 100,000 contour images of different origins and wind directions. Each image in the dataset was a 256 × 256 pixels in size, and we partitioned the entire dataset into 70,000 images for training, 15,000 images for validation, and 15,000 images for testing. The mask images used for training were of the same size as the contour images. Figure 5 shows the test results of the trained model.

### 4.2. Validation

**Figure 6:**
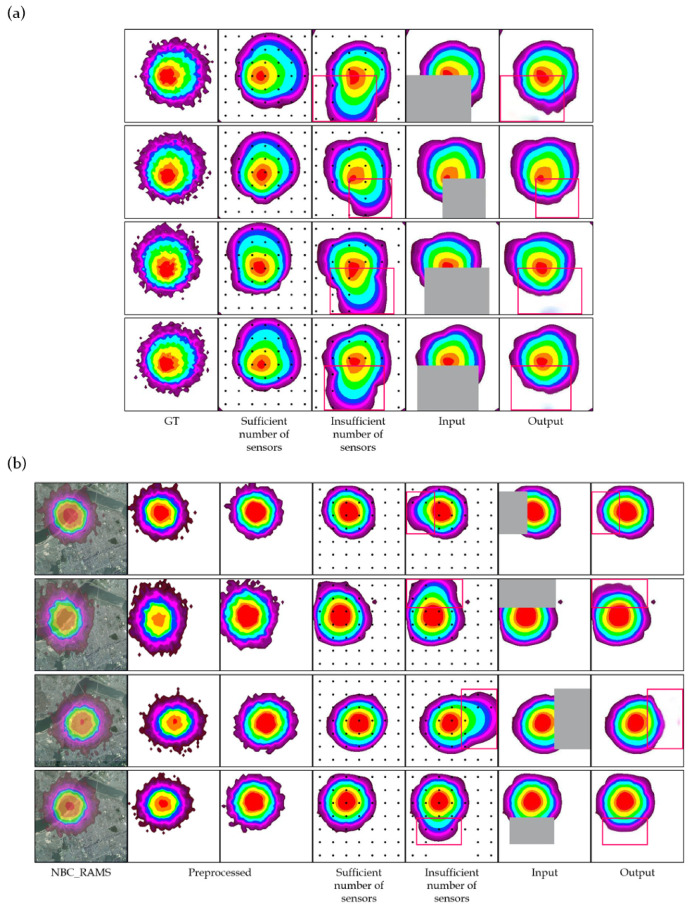
(**a**) The results of validation using the developed diffusion simulation model. From the left, the simulation result; the spatial interpolation results using only the concentration of a sufficient or insufficient number of black dots; contours with the low-reliability parts erased; and the result filled by the model. (**b**) The results of validation using the NBC_RAMS. The image and contour representing the results of NBC_RAMS were added on the left, and an image preprocessed to be suitable for the model is shown in the third column from the left. The remaining images represent the same as in (**a**). The red boxes indicate the regions that were erased and reconstructed.

The validation of the model was conducted using a diffusion simulation model and NBC_RAMS. In each case, the ground truth was the concentration contour generated using the two simulation tools. The concentration values of specific points were sampled from the simulation results, and contours were generated using Kriging interpolation and sampled concentrations. These contours tend to have lower accuracy in areas with sparse data coverage. These areas were erased, and the trained model reconstructed the missing regions. The reconstructed contours were then compared to the ground truth. Significant improvement in the contour accuracy after reconstruction is represented in Figure 6.

#### 4.2.1. Diffusion Simulation Model

The simulation results mimicked an actual environment in which a fatal chemical gas is dispersed. It is assumed that 49 sensors are installed in areas with a sufficient number of sensors and 43 sensors are installed in areas without a sufficient number of sensors. From the simulation results, contour images were generated through spatial interpolation using only the concentrations corresponding to the sensor locations. The accuracy in the areas with relatively few sensors was low, and these areas were erased by the mask and filled with the model. The mIoU score between the restored contours and the simulation results was calculated and compared with the contours before restoration. In order to validate the restoration ability of the model, 50 contours with mIoU scores below 65 were defined as restoration targets. The restoration results showed an average score increase of 13.21%, as listed in Table 1.

#### 4.2.2. NBC_RAMS

The validation was performed using a process similar to that of the diffusion simulation model. The NBC_RAMS results were preprocessed to match the format of the training data. Contours were generated using preprocessed data, and the contours of the environments with an insufficient number of sensors were restored using the model. Compared to the results of the diffusion simulation model, NBC_RAMS demonstrated slightly higher accuracy in environments with a shortage of sensors. In this validation, 50 contours with mIoU scores below 70 were defined as the restoration targets. As presented in Table 2, after the restoration of the model, the average score increased by 11.46%.

#### 4.2.3. Contour Reconstruction with Varied Mask Size

The required mask size for reconstruction varies depending on the extent of inaccurate areas in the spatial interpolation results. In order to evaluate the model’s adaptability to different mask sizes, we classified the masks into three categories: small, medium, and large. Small masks cover 15% or less of the entire image, medium masks cover between 15% and 25%, and large masks cover more than 25%. For each category, the 20 contours generated using the two different simulation tools were used as test data. The results, which are presented in Table 3, demonstrate that the mIoU score increases more significantly with larger masks compared to the spatial interpolation results. However, the reconstruction accuracy decreases as the area to be restored increases.

## 5. Discussion

In this study, we propose a novel method to improve the accuracy of spatial interpolation in gas concentration using image inpainting. By training an image inpainting model with reliable gas diffusion simulation results, we enhanced the accuracy of gas concentration contours by reconstructing the inaccurate regions. The validation using two different simulation tools showed an improvement in accuracy of 13.21% and 11.46% compared to the conventional method: Kriging. The partial convolution model was adopted to reconstruct the erased areas using the valid surrounding values, and the model’s loss function was adjusted to be suitable for gas concentration contour restoration.

Figure 5, which presents the training results, indicates that the model fills in the erased regions of the contour by considering the gas diffusion patterns. The second case from the left shows that the inner region, which was filled based on the surrounding valid areas, closely resembles the ground truth. Additionally, the last case demonstrates that the central regions of the contour are meaningfully filled using the surrounding values. These results suggest that the image inpainting model reconstructs the contour based on gas diffusion patterns rather than merely filling it in a plausible manner.

Figure 6 represents the results of the trained model reconstructing the inaccurate regions within the contour under the assumption of sensor shortage. As the number of sensors decreases, the performance of Kriging interpolation becomes worse. After erasing the unreliable areas of those results, the model reconstructs them, demonstrating improved accuracy. Despite using masks larger than those used for training, the model effectively restores the impaired regions. This demonstrates that partial convolution performs robustly using masks of various sizes, making it suitable for real-world applications.

The proposed model demonstrates notable accuracy improvements in both the validation using simulation tools and the validation using NBC_RAMS. These results indicate that the dataset generated using the simulation tools effectively reflects the gas diffusion patterns. The performance of the proposed model was verified using NBC_RAMS, a verified gas dispersion modeling program. Table 3 represents the results of the model’s adaptability validation for varied mask sizes. When small-sized masks are required, this indicates that the accuracy of spatial interpolation is not significantly low. Therefore, the improvement in accuracy compared to the spatial interpolation results is the lowest. As the mask size increases, the region being reconstructed by the model becomes larger, leading to a greater level of accuracy improvement. However, as the model reconstructs a larger area, the restoration accuracy itself can decrease.

However, the experiments conducted in this study have limitations due to their reliance on simulations. While the model showed good performance in the simulation environment, real-world conditions involve many more variables. In order to minimize the influence of these variables, it is necessary to conduct real gas diffusion experiments and generate concentration contours to compare with the model’s reconstruction results. Therefore, future work may focus on exploring methods to minimize the impact of various variables in real environments. Additionally, the dataset used to train the proposed model does not account for factors such as strong winds or temperature changes due to sunlight, resulting in a lack of diffusion scenarios across various environments. This limitation may introduce biases into the model’s predictions. These biases can be mitigated by developing more accurate simulation tools that consider a wider range of environmental factors. We plan to develop such simulation tools in further studies. Validating the model trained on this enhanced dataset in outdoor environments is expected to significantly improve the model’s reliability.

## 6. Conclusions

A machine learning-based approach is proposed to improve the reliability of spatial interpolation from an image-based perspective using image inpainting. Consecutive numerical values distributed in a specific environment, such as concentration, temperature, and precipitation, make it challenging to determine the overall pattern. The accuracy of estimating the overall environment is greatly influenced by the amount of available data. In real-world situations, if sensors could be installed infinitely, the proposed method would not be necessary, and accurate situation assessment would be possible using only numerical interpolation. However, resources are limited, and it is impossible to install numerous sensors. The proposed method showed performance that exceeds the accuracy of the existing spatial interpolation methods in situations with a limited number of sensors, making it more desirable in terms of resource requirements. Nevertheless, the proposed method requires spatial interpolation as a prerequisite, thereby resulting in higher computational costs and complexity compared to spatial interpolation alone. However, as it is a method for handling 2D image data, the increment in complexity is not significant. In summary, the proposed method is a valuable approach in situations with limited sensor coverage, offering enhanced accuracy over traditional interpolation techniques while managing resource constraints effectively.

Our model focused on gas concentration and reported a meaningful improvement in accuracy through validation using two simulation tools. Chemical gases are colorless and odorless, making it difficult to understand gas diffusion precisely. In order to understand the diffusion of a gas, we must rely on deployed sensors, and a quick response is impossible with a limited number of sensors. In such scenarios, the proposed method can contribute to a more accurate understanding of the extent of diffusion. This approach can be applied not only to gas concentrations and other numerical values. It also provides economic efficiency by enabling sufficiently accurate situation identification using a limited number of sensors. However, for the precise restoration ability of the image inpainting model, it is essential to first develop realistic and dependable simulation tools. The accuracy of image-based interpolation in various fields can be enhanced by training the model using reliable simulation results.

## Figures and Tables

**Table 1:** Validation results using the diffusion simulation model.

mIoU Score		Δ Score
Before Restoration	After Restoration	
60.61%	73.82%	13.21

**Table 2:** Validation results using NBC_RAMS.

mIoU Score		Δ Score
Before Restoration	After Restoration	
68.61%	80.07%	11.46

**Table 3:** Validation results of the model’s adaptability for various mask sizes.

		Size of Mask	
	Small (~15%)	Medium (15~25%)	Large (25%~)
Mean proportion of masked regions	13.88%	20.97%	28.06%
Δ mIoU Score	3.52	6.02	15.14
mIoU Score	79.58%	80.23%	75.57%

## Data Availability

The data presented in this study are available upon request from the corresponding author. The data are not publicly available due to legal restrictions.
